# Isomorphism and similarity for 2-generation pedigrees

**DOI:** 10.1186/1471-2105-16-S5-S7

**Published:** 2015-03-18

**Authors:** Haitao Jiang, Guohui Lin, Weitian Tong, Daming Zhu, Binhai Zhu

**Affiliations:** 1 School of Computer Science and Technology, Shandong University, 1500 Shunhua Road, Jinan, Shandong, 250101, China; 2 Department of Computing Science, University of Alberta, Edmonton, Alberta, T2G 2E6, Germany; 3 Department of Computer Science, Montana State University, Bozeman, MT, 59717, USA

**Keywords:** pedigree similarity, graph isomorphism, NP-complete, FPT algorithms

## Abstract

We consider the emerging problem of comparing the similarity between (unlabeled) pedigrees. More specifically, we focus on the simplest pedigrees, namely, the 2-generation pedigrees. We show that the isomorphism testing for two 2-generation pedigrees is GI-hard. If the 2-generation pedigrees are monogamous (i.e., each individual at level-1 can mate with exactly one partner) then the isomorphism testing problem can be solved in polynomial time. We then consider the problem by relaxing it into an NP-complete decomposition problem which can be formulated as the Minimum Common Integer Pair Partition (MCIPP) problem, which we show to be FPT by exploiting a property of the optimal solution. While there is still some difficulty to overcome, this lays down a solid foundation for this research.

## Introduction

Pedigrees, or commonly known as family trees, are important tools in evolutionary and computational biology. They are important for geneticists, as with a valid pedigree the recombination events can be deduced more accurately [8], or disease loci can be mapped consistently [22,23]. In this sense, pedigrees could greatly help geneticists.

There have been many practical methods for reconstructing pedigrees [30,26,4,5,17]. For instance, Thompson [30] defined the pedigree reconstruction problem as: given the genetic data from a set of extant individuals, reconstruct relationships between the individuals that may share unobserved ancestors. There have also been research using the machine learning methods to construct pedigrees with the maximum likelihood [19,10]. Some theoretical results are also known [27-29].

It is known that a lot of computations on pedigree graphs are NP-hard [24,20,16], so a series of research has been conducted on speeding up these computations [6,13,21]. It is expected that these research will continue, possibly along different directions.

On the other hand, methods for comparing pedigrees are rare. The brute-force method will not work when the data set has size in the thousands [1,14]. People can typically use phylogenetic trees as the basis to compare tree-like pedigrees. On the other hand, even for humans the pedigrees could be more complex than trees as inter-generational mating is not rare. The only known research that systematically study pedigree comparison is by Kirkpatrick *et al*. [18], where the pedigree isomorphism and edit distance problems, for both general pedigrees and leaf-labeled pedigrees, are systemically studied.

In this paper, we follow the work by Kirkpatrick *et al*. [18] to consider the isomorphism and similarity problems for the simplest pedigree -- 2-generation pedigrees, where the isomorphism and similarity problems are both studied. Surprisingly, we show that the isomorphism problem is Gl-hard (GI -- Graph Isomorphism) even for 2-generational pedigrees. We then relax the similarity measure and formulate this as a Minimum Common Integer Pair Partition (MCIPP) problem, generalizing the famous NP-complete Minimum Common Integer Partition (MCIP) problem, which we show to be Fixed-Parameter Tractable (FPT). While these is still some difficulty to overcome, this lays down a solid foundation for this research.

## Preliminaries

An (unlabeled) pedigree is a directed graph *P *= (*I*(*P*), *E*(*P*)) with vertices *I*(*P*) and edges *E*(*P*), together with a gender function *s *: *I*(*P*) → {*male, female*} such that:

1 *P *is acyclic.

2 For all nodes *v *∈ *I*(*P*), the in-degree of *v *is either two or zero.

3 For two edges (*a, c*), (*b, c*) ∈ *E*(*P*), we have *s*(*a*) ≠ *s*(*b*).

**Figure 1:**
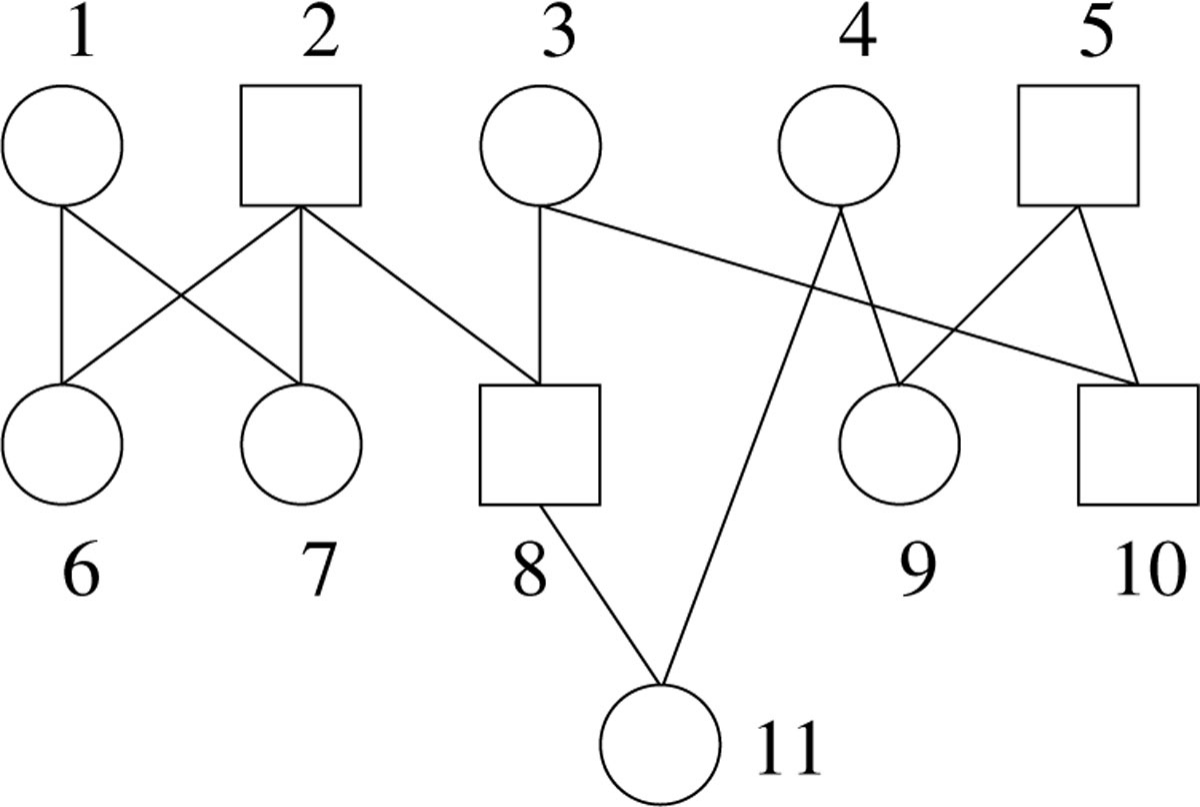
**A simple unlabeled pedigree, the numbers are only used to ease the description**. All edges are downward.

In practice, we typically draw a pedigree in a top-down fashion to denote the direction of the edges. Moreover, we use square (resp. circular) nodes to represent males (resp. females). See Figure 1 for an example. Throughout this paper, we assume that a pedigree (or graph) contains no isolated nodes (i.e., those with in-degree and out-degree both zero). This is easy to handle if it does -- we just remove these isolated nodes.

**Figure 2:**
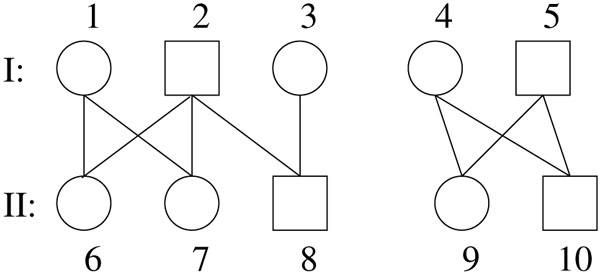
**A 2-generation pedigree, the right component is monogamous**.

Let N={1,2,3,…}. An individual *u *∈ *I*(*P*) is *monogamous *if it mates with exactly one partner, i.e., the number of individuals *u'*, *u' *≠ u, such that (*u, x*), (*u', x*) ∈ *E*(*P*) for some *x *∈ *I*(*P*) is exactly one. A pedigree is *monogamous *if all the individuals are monogamous. In Figure 2, the sub-pedigree formed by the rightmost component is monogamous while the leftmost component is not. A pedigree *P *= (*I*(*P*), *E*(*P*)) is *generational *if there is a function g:I(P)→N such that:

1 *g*(*v*) = 1 for all *v *∈ *I*(*P*) with in-degree zero.

2 For all (*u, v*) ∈ *E*(*P*), we have *g*(*v*) = *g*(*u*) + 1.

The number *g*(*v*) is called the generation of *v*. For a generational pedigree *P*, we use *I_g _*(*P*) to represent the individuals of *P *whose generation is *g*. The pedigree on Figure 1 is not generational, due to node 11. Figure 2 shows a 2-generation pedigree. Throughout this paper, we will focus only on this simplest 2-generation pedigrees.

Given two pedigrees *P *= (*I*(*P*), *E*(*P*)), *P' *= (*I*(*P'*),*E*(*P'*)) with the associated gender functions *s*(−), *s'*(−) respectively, a bijection *ϕ*: *I*(*P*) → *I*(*P'*) is a pedigree isomorphism between *P *and *P' *if:

1 For every *u *∈ *I*(*P*), *s*(*u*) = *s'*(*ϕ*(*u*)), and

2 (*u, v*) ∈ *E*(*P*) if and only if (*ϕ*(*u*), *ϕ*(*v*)) ∈ *E*(*P'*).

## Hardness for 2-generation pedigree

Graph Isomorphism (GI) is one of the most famous problems in computational complexity whose precise complexity has been open since 1972 [15,12]. It is not known to be in P or NP-complete. The class of GI-complete problems are those which are polynomial time equivalent to the GI problem. The class of GI-hard problems are those problems at least as hard as the GI problem. It is known that even testing the isomorphism for chordal bipartite graphs is GI-complete [32].

In [18], it was shown that the pedigree isomorphism problem is GI-hard. The reduction is from bipartite isomorphism. The construction uses a pedigree of three generations. Here we show that even testing the isomorphism of two 2-generation pedigrees is GI-hard.

**Theorem 1 ***Testing the isomorphism between two 2-generation pedigrees is GI-hard*.

*Proof*. We reduce bipartite graph isomorphism problem to our problem. Let *B*_1 _= (*U*_1_, *V*_1_, *E*_1_), *B*_2 _= (*U*_2_, *V*_2_, *E*_2_) be two bipartite graphs (with no isolated nodes). For our construction, we perform the following:

1 All nodes in *U*_1_,*U*_2 _are marked male.

2 All nodes in *V*_1_,*V*_2 _are marked female.

3 *B*_1 _is converted into a pedigree *P*_1 _= (*I*(*P*_1_), *E*(*P*_1_)) as follows: (3.1) *I*(*P*_1_) = *U*_1 _∪ *V*_1 _are the generation-1 nodes; (3.2) *E*(*P*_1_) is initially set as empty; (3.3) for (*u, v*) ∈ *E*_1 _we create a new generation-2 node *uv *such that *s*(*uv*) = *female, E*(*P*_1_) ← *E*(*P*_1_) ∪ {(*u*, *uv*), (*v, uv*)}.

4 *B*_2 _is converted into a pedigree *P*_2 _identically as in step (3).

We claim that *B*_1 _and *B*_2 _are isomorphic iff *P*_1 _and *P*_2 _, both 2-generational, are isomorphic. We only show the necessary direction here as the other one is easy. If *P*_1 _and *P*_2 _are isomorphic, the first property we make use of is that all the generation-2 nodes are female. So, in the isomorphism between *P*_1 _and *P*_2_, if a generation-2 node *uv *∈ *I*(*P*_1_) is mapped to a generation-2 node *xy *∈ *I*(*P*_2_), we can simultaneously contract *uv, xy *to their corresponding male parents in *P*_1 _and *P*_2_. Consequently, we obtain the isomorphism between *B*_1 _and *B*_2_.   □

**Figure 3:**
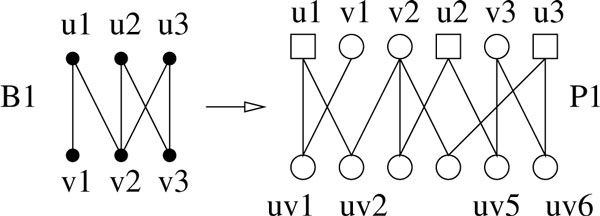
**An example for the GI-hardness reduction**. In *P*_1_, only four (the leftmost and rightmost two) generation-2 nodes are labeled, some underlined and some overlined, to maintain clarity.

Note that in our construction, all generation-2 individuals are female; moreover, a pair of generation-1 individuals mate with exactly one female child. A simple example on this reduction is shown on Figure 3.

Although the isomorphism testing problem is GI-hard even for 2-generation pedigrees, in some situations the problem is not hard to solve. In fact, when both of the 2-generation pedigrees are monogamous then the problem can be solved in linear time.

When a pair of generation-1 couple mate to have generation-2 children, for instance *i *females and *j *males, we say that these two parents and the *i *+ *j *children form an 〈*i*, *j*〉-family. In Figure 2, the rightmost component is a 〈1,1〉-family.

**Theorem 2 ***Testing the isomorphism between two 2-generation monogamous pedigrees is polynomial time solvable*.

*Proof*. It is easily seen that when a 2-generation pedigree *Q*_1 _is monogamous then it is composed of a set of disjoint 〈*i*, *j*〉-families. So to test the isomorphism between two monogamous 2-generation pedigrees *Q*_1_,*Q*_2 _it suffices to check whether two sets of integral pairs are identical, which can be done in *O*(*n *log *n*) time using the standard optimal sorting algorithms in two passes similar to the radix sort. In the first pass, we sort all the pairs according to their first components, and in the second, for each contiguous list of pairs with the same first component, we sort them according to the second components.   □

## Similarity of 2-generation pedigrees

The hardness result in the previous section implies that it might be too much if we use the standard isomorphism to measure the similarity of 2-generation pedigrees. In practice, ambiguities exist in pedigree-related datasets. In fact, it is estimated that 2-10% of people do not know their biological father [2,25]. For 2-generation pedigrees, in general the pedigrees cannot be monogamous. So, we need a new measure to weakly describe the similarity of two 2-generation pedigrees.

For a general 2-generation pedigree *P*, it is not difficult to identify all (not necessarily disjoint) 〈*i*, *j*〉-families (or simply families, when 〈*i*, *j*〉's are used). (For instance, the left component in Figure 2 can be decomposed into two families: 〈2, 0〉 and 〈0, 1〉.) Then, we try to decompose the generation-2 nodes in these families so that the resulting number of isomorphic sub-families is minimized. Note that in this process a generation-1 pair can appear in more than one sub-family. This can in turn be formulated as the Minimum Common Integer Pair Partition (MCIPP) problem.

### MCIP and MCIPP Problems

Throughout this paper, for MCIP, we focus on integers in N={1,2,3,…}. A partition of an integer *n *is a multiset *τ*(*n*) = {*n*_1_, *n*_2_,..., *n_t_*} such that $\sum_{1\leq i\leq t}n_{i}=n$. For example, when *n *= 9, {1, 2, 2, 4} is a partition of *n*. It should be noted that while it is simple to partition an integer, the number of such partitions is usually (counter-intuitively) huge. For instance, the integer 10 has 190569292 distinct partitions [3].

A partition of a multiset *X *= {*x*_1_, *x*_2_,...,*x_p_*} is a multiset union of all the partitions *τ*(*x_i_*), i.e., ∪_1≤*i*≤*p*_*τ*(*x_i_*). A multiset *Z *is a common partition of two multisets *X *= {*x*_1_, *x*_2_,..., *x_p_*}, *Y *= {*y*_1_, *y*_2_,..., *y_q_*} if there are partitions *τ*_1_, *τ*_2 _with ∪_1≤*i*≤*p*_*τ*_1_(*x_i_*) = ∪_1≤*j*≤*q*_*τ*_2_(*y_j_*) = *Z*. The size of the partition *Z *is denoted as |*Z*|. For example, given *X *= {5, 8}, *Y *= {3,10}, a common partition of *X*, *Y *is *Z *= {1, 2, 2, 4, 4}, and the size of this partition is 5. It is easily seen that the necessary condition for *X *and *Y *to admit a common partition is that the sums of the integers in *X *and *Y *are equal. Throughout this paper, whenever we talk about a common partition for sets of integers *X *and *Y*, we always assume that this condition is met.

#### MCIP (Minimum Common Integer Partition)

Instance: Two multiple sets of integers *A *and *B*, and an integer *k*.

Question: Does *A*, *B *admit a common partition of size *k*?

For the ease of presentation, we use MCIP(A, B) to represent this instance.

Given a 2-tuple of integers, 〈*a, b*〉, the projection $P_{1}\left(\langle a,b\rangle \right)=a,P_{2}\left(\langle a,b\rangle \right)=b$. Let *S *be a set of 2-tuples of integers, $P_{1}\left(S\right)=\cup_{s\in S}P_{1}\left(s\right),P_{2}\left(S\right)=\cup_{s\in S}P_{2}\left(s\right)$.

Given two sets of 2-tuples *S*, *T*, a common partition of *S *and *T *is a set of 2-tuples *H *= {〈*g*_1_, *h*_1_〉, 〈*g*_2_, *h*_2_〉, ⋯, 〈*g_k_*, *h_k_*〉} such that $P_{1}\left(H\right)$ is a common partition of $P_{1}\left(S\right)$ and $P_{1}\left(T\right)$, and, $P_{2}\left(H\right)$ is a common partition of $P_{2}\left(S\right)$ and $P_{2}\left(T\right)$. *k *is the size of the partition *H*. Again, it is easily seen that the necessary condition for *S *and *T *to admit a common partition is that the sums of the integers in $P_{1}\left(S\right)$ and $P_{1}\left(T\right)$ are equal, so are those in $P_{2}\left(S\right)$ and $P_{2}\left(T\right)$. Throughout this paper, whenever we talk about any common partition of sets of 2-tuples *S, T*, we always assume that this condition is met.

#### MCIPP (Minimum Common Integer Pair Partition)

Instance: Two multiple sets of 2-tuples of integers *S *and *T*, and an integer *k*.

Question: Does *S, T *admit a common partition of size *k*?

Recall that a 2-tuple 〈*i, j*〉 represents the pedigree of a couple which has *i *female and *j *male chilren. Again, we use MCIPP(S, T) to represent this instance. As MCIPP is a generalization for MCIP, all the known negative results regarding MCIP hold for MCIPP; i.e., MCIP and MCIPP are both NP-complete and APX- hard, following [7]. (In the past, *d*-MCIP has also been considered, where the input is *d *multisets with the same sum. Efficient asymptotic approximation algorithms have been obtained for large *d *[7,33,34], the best factor being 0.5625 · *d *+ O(1) [34]. We will only consider *d *= 2 in this paper.) Also, note that the integer 0 in a solution for MCIP is meaningless while it is possible that 0 can appear either in the input or in the solution for MCIPP. So for MCIPP, we focus on integers in N∪{0}={1,2,3,…}.

Finally, a Fixed-Parameter Tractable (FPT) algorithm is an algorithm for a decision problem with input size *n *and parameter *k *whose running time is *O*(*f *(*k*)*n^c^*) = O*(*f*(*k*)), where *f *(−) is any computable function on *k *and *c *is a constant. FPT algorithms are efficient tools for handling some NP-complete problems, especially when *k *is small in practical datasets [9,11].

### Some properties of MCIPP

Given a pair of integers *a, c*, we say *a dominates c *if *a *>*c*. Given a pair of 2-tuples of integers 〈*a, b*〉 and 〈*c*, *d*〉, we say 〈*a, b*〉 *dominates *〈*c*, *d*〉 if *a *≥ *c *and *b *≥ *d*. To simplify the writing, we say that 〈*a, b*〉 and 〈*c*, *d*〉 form a *dominating pair *if either 〈*a, b*〉 *dominates *〈*c*, *d*〉 or vice versa. Likewise, 〈*a, b*〉 and 〈*c*, *d*〉 form a *non-dominating pair *if either *a *>*c*, *b *<*d *or *a *<*c*, *b *>*d*.

We first describe some optimality properties for both the optimization versions of MCIP and MCIPP. When the context is clear, we still use MCIP(-,-) and MCIPP(-,-) to denote the corresponding optimization versions of the instances.

**Lemma 1 ***Let A, B be the input for MCIP. In any feasible solution, if a partition for some × ∈ A, τ (x) = *{*x*_1_, *x*_2_,...,*x_p_*}, *and a partition for some y *∈ *B, tau*(*y*) = {*y*_1_, *y*_2_,...,*y_q_*}, *satisfies that |τ*(*x*) ∩ *τ*(*y*)| > 1 *then this solution for MCIP is not optimal*.

*Proof*. Suppose to the contrary that |*τ*(*x*) ∩ *τ*(*y*)| > 1, and the corresponding partition for *A, B *is optimal. WLOG, suppose *τ*(*x*) = {*x*_1_, *x*_2_,..., *x_p_*} and *τ*(*y*) = {*y*_1_, *y*_2_,..., *y_q_*} contain *r *common elements {*z*_1_, *z*_2_,..., *z_r_*} then we can update *τ*(*x*) ← *τ*(*x*) − {*z*_1_, *z*_2_,..., *z_r_*}∪{*z*_1 _+ *z*_2 _+ ... + *z_r_*} and *τ*(*y*) ← *τ*(*y*) − {*z*_1_, *z*_2_,..., *z_r_*}∪{*z*_1 _+ *z*_2 _+ ... + *z_r_*}. Then the solution size for MCIP on *A, B *is reduced by *r *− 1, contradicting the optimality of the assumption.   □

With the above lemma, we can now assume that for any optimal partition for some *x *∈ *A *and some *y *∈ *B*, they share at most one common element. Notice that this lemma also holds for MCIPP, i.e., in an optimal partition of 〈*s*_1_, *s*_2_〉 ∈ *S *and 〈*t*_1_, *t*_2_〉 ∈ *T*, *τ*(〈*s*_1_, *s*_2_〉) and *τ*(〈*t*_1_, *t*_2_〉) share at most one common 2-tuple. Similarly, we can assume that in the input for *MCIP *(*A, B*) (resp. *MCIPP *(*S, T*)) there is no common pair of integers in *A *and *B *(resp. no common pair of 2-tuples in *S *and *T*), as it must be put in the optimal solution.

The following property is trivial and holds for both MCIP and MCIPP.

**Lemma 2 ***Let *|MCIPP*(*S, T*)| *be the optimal solution size for MCIPP*(*S, T*). *Then *|MCIPP*(*S, T*)| >*max{|S|, |T|}*.

For a pair of dominating 2-tuples 〈*a, b*〉 and 〈*c*, *d*〉, we can use subtraction to partition them into two common pairs. For example, if *a *≤ *c *and *b *≤ *d*, then we can obtain the common partition {〈*a, b*〉, 〈*c *− *a, d *− *b*〉. So, given 〈2, 4〉 and 〈4, 5〉 we can obtain a partition {〈2,4〉, 〈2,1〉} for 〈4, 5〉. We also say that this is a *dominating - partition *operation. Apparently, for MCIP, this gives a way to partition a pair of integers as well. For instance, given 2 and 6, we can subtract 2 from 6 to obtain a partition {2, 4} for 6.

We next describe some properties on non-dominating pairs of 2-tuples which are unique for MCIPP -- for MCIP, a pair of integers *a, b *has the property that either *a *dominates *b *or vice versa. This is not the case for a non-dominating pairs of 2-tuples, e.g. 〈1, 4〉 and 〈2, 3〉. We start with this fundamental lemma.

**Lemma 3 ***Let A', B' be a set of positive integers with the same total sum, moreover, let us suppose |A'| = m *+ 1, *|B'| = m. Then there must exist elements a *∈ *A', b *∈ *B' such that a *<*b*.

*Proof*. As |*A'*| > |*B'*|, we can arbitrarily select *m *elements from *A' *and match up them with those in *B *in an one-to-one fashion. As the sum of integers in *A *and *B *are the same, in this matching at least one of elements *a *∈ *A *must be smaller than its matched counterpart *b *∈ *B *-- otherwise, the sum of integers in *A *would be larger than that of *B'*.   □

**Corollary 1 ***Let A, B be two sets of n *> 1 *positive integers with the same total sum. WLOG, let A = {a*_1_, *a*_2_, ⋯, *a_n_*}, *B *= {*b*_1_, *b*_2_, ⋯, *b_n_*}. *Then there must exist an element b *∈ *B' = B *− {*b_j_*} *which is greater than some element a *∈ *A' *= {*a*_1_, *a*_2_, ⋯, *a*_*i*−1_, *a_i _*− *b_j_*, *a*_*i*+1_, ⋯, *a_n_*}, *where a_i _*>*b_j_*.

*Proof*. Obviously we have |*A'*| = *n *and |*B'*| = *n *− 1. Then this corollary follows directly from Lemma 3.   □

The implication of Corollary 1 for MCIP with input *A, B *is obvious -- we can successively find pairs of dominating integers. In fact, in the proof of Corollary 1, once we obtain *a' *= *a_k _*∈ *A' *and *b' *= *b_ℓ _*∈ *B' *such that *a' *<*b'*, we can repeatedly use the above argument to *A'' *= *A' *− {*a'*} = {*a*_1_, *a*_2_, ⋯, *a*_*k*−1_, *a*_*k*+1_, ⋯, *a_n_*} and *B'' *= {*b*_1_, *b*_2_, ⋯, *b*_ℓ−1_, *b*_ℓ _− *a', b*_ℓ+1_, ⋯, *b*_*n*_}, where |*A''*| = |*B''*| = *n *− 1 and the two sets *A, B *have the same sum.

Now let us see how this can be applied to MCIPP. When we have an instance of MCIPP whose input {*S, T*} is each composed of *m *non-dominating 2-tuples, then we can find a pair *s *= 〈*s*_1_, *s*_2_〉 ∈ *S *and *t *= 〈*t*_1_, *t*_2_〉 ∈ *T *(assuming *s*_1 _>*t*_1 _and *s*_2 _<*t*_2_) such that we can put 〈*t*_1_, *s*_2_〉 in some solution set while the resulting instance *S*' = ({*S *− {〈*s*_1_, *s*_2_〉}) ∪ {〈*s*_1 _− *t*_1_, 0〉},*T' *= ({*T *− {〈*t*_1_, *t*_2_〉}) ∪ {〈0, *t*_2 _− *s*_2_〉} is still a valid instance for MCIPP. (We call this operation *non-dominating-partition*.) Then, following Corollary 1, there exists a pair of dominating 2-tuples *s' *∈ *S', t' *∈ *T'*. Moreover, if we apply the dominating-partition process on these two tuples *s', t'*, following Corollary 1, we can repeatedly find dominating tuples until all tuples in *S, T *are all commonly partitioned. This is because after we apply the dominating-partition on *s', t' *(say *s' *<*t'*) to obtain an MCIPP instance *S'',T''*, we have $\left|P_{1}\left(S^{\prime \prime }\right)|\neq |P_{1}\left(T^{\prime \prime }\right)|,|P_{2}\left(S^{\prime \prime }\right)|\neq |P_{2}\left(T^{\prime \prime }\right)\right|$ and the two pairs of sizes in fact differ by one. Following Corollary 1, we can then repeatedly obtain dominating pairs.

*Algorithm Heuristic-MCIPP*(*S, T*)

Input: *S*, *T*

Output: A common partition *τ*(*S*, *T*) for *S*, *T*, initially empty.

1   While |*S*| ≥ 2 and |*T*| ≥ 2

2      Repeat

2.1         select a pair of dominating 2-tuples, *s *∈ *S *and *t *∈ *T*,

2.2         compute two decomposing 2-tuples by subtraction,

2.3         update *S *← *S *− {*s*}, *T *← (*T *− {*t*}) ∪{*t *− *s*} if *s *<*t*,

2.4         update *S *← (*S *− {*s*}) ∪{*s *− *t*}, *T *← *T *− {*t*} if *s *>*t*,

2.5         update *τ*(*S*, *T*) ←*τ*(*S*, *T*) ∪ {min(*s*, *t*)},

3      Until no dominating 2-tuples can be found.

4      If there are at least two pairs of non-dominating 2-tuples in *S *and *T*

5      Then

5.1         use a brute-force method to select two non-dominating 2-tuples *s' *∈ *S*, *t' *∈ *T *which leads to successive dominating pairs.

6   If |*S*| = 1, |*T*| = 1, then find the smaller tuple in *S *and *T*, *x*.

7   Return *τ*(*S*, *T*) ← *τ*(*S*, *T*) ∪ {*x*}.

Of course, due to the 'existence' constraint in Lemma 3 and Corollary 1, we would have to use a brute-force method to find a pair *s *∈ *S, t *∈ *T *which can make the process of repeatedly processing dominating pairs possible. Let us show an example, *S *= {〈9, 4〉, 〈1, 11〉, 〈6, 3〉} and *T *= {〈2, 8〉, 〈12, 1〉, 〈2, 9〉}. In this example, among the 9 non-dominating pairs between *S *and *T*, there are 4 solutions enabling us to successively find dominating pairs. One of them is *s *= 〈6, 3〉 and *t *= 〈2, 9〉, which gives us a common partition of size 6. The other 5 solutions all lead to a common partition of size 7.

The above discussion enables us to design an algorithm Heuristic-MCIPP to prove the next lemma.

**Lemma 4 ***Let *|*MCIPP*(*S, T*)| *be the size of the solution returned by Heuristic-MCIPP. Then *|*MCIPP*(*S, T*)| ≤ |*S*| + |*T*|.

*Proof*. When there is no non-dominating pairs in the input, with the running of the algorithm Heuristic-MCIPP, we have |*MCIPP*(*S*, *T*)| ≤ |*S*| + |*T*| − 1. The reason is that when each of *S *and *T *has at least two 2-tuples, we can use the dominating-partition procedure to obtain two 2-tuples in the solution set for each pair of dominating 2-tuples from *S, T*. When there are a total of three elements in *S, T*, say, one in *S *and two in *T*, we just need to return the two elements in *T *as their sum matches the one in *S *already. (This is certainly true for MCIP as pointed out in [7].)

When there are *p *≥ 2 pairs of non-dominating pairs at Step 4-5 of the Heuristic-MCIPP algorithm, following Corollary 1 and the subsequent arguments, there exists a non-dominating pair *s *= 〈*s*_1_, *s*_2_〉 ∈ *S*, *t *= 〈*t*_1_, *t*_2_〉 ∈ *T *which leads to successive dominating pairs. (We can use the brute-force method to find this in *O*(*p*^2^(|*S*| + |*T*|)) time.) In this case, the solution obtained by Heuristic-MCIPP has size at most 1 + (|*S*| + |*T*| − 1) = |*S*| + |*T*|, where the first one corresponds to (*s*_1_, *t*_2_) (if *s*_1 _<*t*_1_) or (*t*_1_, *s*_2_) (if *s*_1 _>*t*_1_).   □

In fact, the above three lemmas imply that Heuristic-MCIPP provides a factor-2 approximation for MCIPP, as we have |*S*| + |*T*| ≤ 2max{|*S*|, |*T*|} ≤ 2|MCIPP*(*S, T*)|. On the other hand, designing approximation algorithms is not our focus for this paper; in fact, by a simple modification for the Maximum Packing method in [7] we can obtain a similar factor-1.25 approximation for MCIPP. In the remainder of this paper, we solely focus on the exact or FPT algorithm.

Note that Lemma 4 is different from its counterpart for MCIP, which, according to Lemma 2.2 in [7], states that |*MCIP*(*A, B*)*| *< |*A*| + |*B*| − 1. The latter in fact immediately implies that for MCIP there is always an optimal solution which does not partition at least an integer from either *A *or *B*. That further implies that there is a simple FPT algorithm for MCIP based on the bounded-degree search. We will show a stronger property in the next section to improve the FPT algorithm for MCIP, and subsequently, an FPT algorithm for MCIPP can be obtained.

### An FPT algorithm for MCIPP

We first give the following lemma for MCIP.

**Lemma 5 ***Let A, B be the input for MCIP and let a be the smallest element in A or B. Then there is an optimal solution for MCIP which contains a, i.e., there is an optimal solution which does not partition the smallest element in A and B*.

*Proof*. We first show the following claim: in an optimal solution *τ *for MCIP with input *A, B*, let *a*_1_, *a*_2 _be a pair of elements in *τ*(*z*), *z *∈ *A *∪ B, with the condition that (1) *a*_1 _+ *a*_2 _<*a*, and (2) *a*_2 _is the minimum among all pairs of elements in *τ *satisfying the condition (1), then there is an optimal solution *τ' *which partitions some element *z *∈ *A *∪ *B *with *τ'*(*z*) = *τ*(*z*) − {*a*_1_, *a*_2_} ∪ {*a*_1 _+ *a*_2_}.

The proof for the above claim is as follows. WLOG, let *z *∈ *A *and let *a*_1 _∈ *τ*(*y*_1_) and *a*_2 _∈ *τ*(*y*_2_) for two distinct integers *y*_1_, *y*_2 _∈ B. Following the definition of 〈*a*_1_, *a*_2_〉, *τ*(*y*_1_) contains at least one more element other than *a*_1_, say *a*_3_; and following (2) we have *a*_3 _>*a*_2_. Suppose that *a*_3 _∈ *τ*(*x*) for some *x *∈ *A*. By Lemma 1, *x *≠ *z*. We replace *a*_3 _by $a_{3}^{\prime }=a_{3}-a_{2}$, and *a*_1 _by $a_{1}^{\prime }=a_{1}+a_{2}$. Subsequently, we obtain another optimal partition *τ' *with *τ'*(*x*) = *τ*(*x*) − {*a*_3_} ∪ {$a_{3}^{\prime }$, *a*_2_}, *τ'*(*y*_1_) = *τ*(*y*_1_) − {*a*_1_, *a*_3_} ∪ {$a_{1}^{\prime }$, $a_{3}^{\prime }$}, and *τ'*(*z*) = *τ*(*z*) − {*a*_1_, *a*_2_} ∪ {$a_{1}^{\prime }$}. Apparently, *τ' *has the same size as *τ*, so it is also a minimum size common partition for *A, B*.

It is obvious that, as long as the smallest element *a *is partitioned in some optimal partition *τ *with $\tau \left(a\right)=\left\{a_{1}^{\prime },a_{2}^{\prime },\ldots ,a_{t}^{\prime }\right\}$, we can repeatedly apply the above steps to obtain another optimal partition *τ' *with $\tau^{\prime }\left(a\right)=\left\{a_{1}^{\prime },a_{2}^{\prime },\ldots ,a_{i-1}^{\prime },a_{i}^{\prime }+a_{j}^{\prime },a_{i+1}^{\prime },\ldots ,a_{j-1}^{\prime },a_{j+1}^{\prime },\ldots ,a_{t}^{\prime }\right\}$. After *t *− 1 such steps, we obtain an optimal partition which contains the minimum element *a *in *A *∪ *B*.   □

An example of the above proof is given as follows. We have *A *= {2, 5, 5}, *B *= {6, 6}, and an optimal partition *τ *= {1,1, 5, 5} where *τ*(2) = {*a*_1 _= 1, *a*_2 _= 1}. By the construction in the proof of Lemma 5, *y*_1 _= 6, *y*_2 _= 6, *a*_3 _= 5, $a_{3}^{\prime }=4$ and $a_{1}^{\prime }=2$. The new optimal solution is *τ' *= {1, 2, 4, 5}, where *a *= 2 is kept.

We comment that we can use Lemma 2.2 by Chen *et al*. [7] directly to prove a weaker claim: as |*MCIP*(*A*, *B*)| < |*A*| + |*B*| − 1, there must be an optimal solution whose corresponding matching graph between the partitioned elements in *A, B *contains no cycle, which means there is at least one leaf node. Then this leaf node corresponds to an unpartitioned integer in *A *or *B*. The above lemma in fact implies a faster FPT algorithm for MCIP. Pick the smallest element *a *∈ *A *∪ *B *(say *a *∈ *A*), we try to partition some other integer *z *∈ *B *by subtracting *a *from it. Then we repeat over the new problem instance involving *z *− *a*. This process is repeated *k *times when either a solution is founded or we have to report that there is no solution of size *k*. The running time is O*((max{|*A*|, |*B*|})*^k^*) = O*(*k^k^*).

To obtain an FPT algorithm for MCIPP, we also need a similar lemma.

**Lemma 6 ***Let S, T be the input for MCIPP. Then there is an optimal solution for MCIPP which either contains *〈*a, b*〉 ∈ *S *∪ *T or *〈*c, d*〉 ∈ *S *∪ *T, or contains *〈*a, d*〉, *where a is the minimum element in *$P_{1}\left(S\cup T\right)$*and d is the minimum element in *$P_{2}\left(S\cup T\right)$.

*Proof*. Again, we first show the following claim: in an optimal solution *τ *for MCIPP with input *S, T*, let 〈*a*_1_, *a*_2_〉, 〈*b*_1_, *b*_2_〉 be two 2-tuples in *τ*(*z*), *z *∈ *S *∪ *T*, such that (1) *a*_1 _+ *b*_1 _≤ *a*, and (2) *b*_1 _is the minimum among all pairs of 2-tuples in *τ *satisfying (1), then there is an optimal solution *τ' *which partitions some 2-tuple *z *∈ *S *∪ *T *with *τ'*(*z*) = *τ*(*z*) − {〈*a*_1_, *a*_2_〉, 〈*b*_1_, *b*_2_〉} ∪ { *a*_1 _+ *b*_1_, *a*_2_}. (Symmetrically, we can have a claim on the second component of 2-tuples in *S *∪ *T*, i.e., *d*.)

WLOG, let *z *= 〈*z*_1_, *z*_2_〉 ∈ *S *and let 〈*a*_1_, *a*_2_〉 ∈ *τ*(*y*_1_) and 〈*b*_1_, *b*_2_〉 ∈ *τ*(*y*_2_) for two distinct 2-tuples *y*_1_, *y*_2 _∈ *T*. Following the definition of (*a*_1_, *b*_1_), *tau*(*y*_1_) contains at least one more pair 〈*c*_1_, *c*_2_〉, with *c*_1 _≥ *b*_1_. Suppose that 〈*c*_1_, *c*_2_〉 ∈ *τ*(*x*) for some *x *∈ *S*. Again, by Lemma 1, *x *≠ *z*. We replace 〈*c*_1_, *c*_2_〉 by 〈*c*_1 _− *b*_1_, *c*_2_〉, and 〈*a*_1_, *a*_2_〉 by 〈*a*_1 _+ *b*_1_, *a*_2_〉. Subsequently, we obtain another optimal partition *τ' *with *τ'*(*x*) = *τ*(*x*) − {〈*c*_1_, *c*_2_〉}∪{〈*c*_1 _− *b*_1_, *c*_2_〉, 〈*a*_2_, *b*_2_〉}, *τ'*(*y*_1_) = *τ*(*y*_1_) − {〈*a*_1_, *a*_2_〉, 〈*c*_1_, *c*_2_〉 ∪ {〈*a*_1 _+ *b*_1_, *a*_2_〉}, 〈*c*_1 _− *b*_1_, *c*_2_〉, and *τ'*(*z*) = *τ*(*z*) − {〈*a*_1_, *a*_2_〉, 〈*b*_1_, *b*_2_〉 ∪ {〈*a*_1 _+ *b*_1_, *a*_2_〉}. Again, *τ' *is also a minimum size common partition for *S, T*.

Similar to Lemma 5, it is obvious that we can repeatedly apply the above steps to obtain an optimal solution with does not partition the smallest element in $P_{1}\left(S\cup T\right)$ (and, symmetrically, $P_{2}\left(S\cup T\right)$). Hence the lemma is proven.   □

With the above lemma, it is again possible to have an FPT algorithm, Exact-MCIPP, for MCIPP using bounded degree search. At each step, we search for 〈*a, b*〉, 〈*c, d*〉 ∈ *S *∪ *T *or 〈*a, d*〉 ∈ *S *∪ *T*, where *a *is the minimum element in $P_{1}\left(S\cup T\right)$ and *d *is the minimum element in $P_{2}\left(S\cup T\right)$ such that some optimal solution for MCIPP contains 〈*a*, *b*〉, 〈*c, d*〉 or 〈*a*, *d*〉. For one step, the running time for the former would be *O*(*k*_1 _+ *k*_2_) for the first two cases and for the latter would also be *O*(*k*_1 _+ *k*_2_) -- as 〈*a, d*〉 could be subtracted from *O*(*k*_1 _+ *k*_2_) pairs, where *k*_1 _= |*S*|, *k*_2 _= |T|. As *k*_1_, *k*_2 _≤ *k*, the running time of this step is bounded by *O*(2*k*). Running this for *k *steps, the running time of the whole algorithm is O*(2*^k^k^k^*). Hence, we have the following theorem.

Algorithm *Exact-MCIPP(S, T)*

Input: *S, T, k*

Output: A common partition *τ*(*S*, *T*) for *S*, *T*, initially empty.

1   While *k *≥ 1

2      Repeat

2.1         let *a *be the minimum element in $P_{1}\left(S\cup T\right)$,

2.2         let *d *be the minimum element in $P_{2}\left(S\cup T\right)$,

2.3         if 〈*a*, *d*〉 ∈ *S *∪ *T *then *τ*(*S*, *T*) ← *τ*(*S*, *T*) ∪ {〈*a*, *d*〉}, delete 〈*a*, *d*〉 from *S *∪ *T*, and update *S*, *T *and *k *← *k *− 1,

2.4         if 〈*a*, *b*〉 ∈ *S *∪ *T *then *τ*(*S*, *T*) ← *τ*(*S*, *T*) ∪ {〈*a*, *b*〉}, delete 〈*a*, *b*〉 from *S *∪ *T*, and update *S*, *T *and *k *← *k *− 1,

2.5         if 〈*c*, *d*〉 ∈ *S *∪ *T *then *τ*(*S*, *T*) ← *τ*(*S*, *T*) ∪ {〈*c*, *d*〉}, delete 〈*c*, *d*〉 from *S *∪ *T*, and update *S*, *T *and *k *← *k *− 1,

3      Until *S *= ∅ or *T *= ∅ or *k *= 0.

4   If both *S *= ∅ and *T *= ∅

4.1      Then return *τ*(*S*, *T*),

4.2      Else return 'no solution'.

**Theorem 3 ***Minimum Common Integer Pair Partition is FPT*.

The running time of the above FPT algorithm is still too high to be applied alone to the similarity comparison for arbitrary 2-generation pedigrees, i.e., when *k *is large. In [14], the salmon data contains 60 individuals from each family, with hundreds of families. To handle some data like that, we either need to speed up the running time of our algorithm or combine the FPT algorithm with some existing approximation algorithms (which will be discussed next). Nevertheless, it lays down a solid theoretical foundation for further research on this problem, especially when *k *is relatively small.

In practice, to handle datasets possibly of varying *k *values, we suggest a combination of the FPT algorithm and approximation algorithms [7,31]. That is, when the value of *k *is not too large, we can run this FPT algorithm; when *k *is too large for the FPT algorithm to handle, we can then use the approximation algorithms. (We comment that the approximation algorithms in [7,31], though presented for MCIP, can be easily adapted for MCIPP.)

## Concluding remarks

We consider the problem of testing the isomorphism and similarity of the simplest possible unlabeled pedigrees. We show that the isomorphism testing is GI-hard, excluding any chance for a polynomial time algorithm (unless Graph Isomorphism is polynomially solvable). We define a new similarity measure based on 〈*i, j*〉-family, and formulate this as the Minimum Common Integer Pair Partition (MCIPP) problem, which generalizes the NP-complete problem of Minimum Common Integer Partition (MCIP) problem. We show that MCIPP (hence MCIP) is FPT (Fixed-Parameter Tractable). It would be interesting to significantly improve the running time of the FPT algorithms presented in this paper.

## Competing interests

The authors declare that they have no competing interests.

## Authors' contributions

BZ conceived the study. All authors contributed to the algorithm design and analysis, read and approved the final manuscript.

## Declarations

Publication charges for this work was funded by NSF of China grant 61202014.

This article has been published as part of *BMC Bioinformatics *Volume 16 Supplement 5, 2015: Selected articles from the 10th International Symposium on Bioinformatics Research and Applications (ISBRA-14): Bioinformatics. The full contents of the supplement are available online at http://www.biomedcentral.com/bmcbioinformatics/supplements/16/S5.
